# Loss of complement regulatory proteins on uninfected erythrocytes in vivax and falciparum malaria anemia

**DOI:** 10.1172/jci.insight.124854

**Published:** 2018-11-15

**Authors:** Damian A. Oyong, Enny Kenangalem, Jeanne R. Poespoprodjo, James G. Beeson, Nicholas M. Anstey, Ric N. Price, Michelle J. Boyle

**Affiliations:** 1 Menzies School of Health Research and; 2 Charles Darwin University, Darwin, North Territory, Australia.; 3 Mimika District Health Authority and; 4 Papuan Health and Community Development Foundation, Timika, Papua, Indonesia.; 5 Department of Paediatrics, Faculty of Medicine, Gadjah Mada University, Yogyakarta, Central Java, Indonesia.; 6 Burnet Institute, Melbourne, Victoria, Australia.; 7 Department of Medicine, University of Melbourne, Melbourne, Victoria, Australia.; 8 Department of Microbiology and Central Clinical School, Monash University, Melbourne, Victoria, Australia.; 9 Centre for Tropical Medicine and Global Health, Nuffield Department of Clinical Medicine, University of Oxford, Oxford, United Kingdom.

**Keywords:** Immunology, Infectious disease, Complement, Malaria

## Abstract

Anemia is a major complication of malaria, driven largely by loss of uninfected RBCs during infection. RBC clearance through loss of complement regulatory proteins (CRPs) is a significant contributor to anemia in *Plasmodium falciparum* infection, but its role in *Plasmodium vivax* infection is unknown. CRP loss increases RBC susceptibility to macrophage clearance, a process that is also regulated by CD47. We compared CRPs and CD47 expression on infected and uninfected RBCs in adult patients with vivax and falciparum malaria and different anemia severities from Papua, Indonesia. Complement activation and parasite-specific complement-fixing antibodies were measured by ELISA. Levels of CR1 and CD55 were reduced in severe anemia in both falciparum and vivax malaria. Loss of CRPs and CD47 was restricted to uninfected RBCs, with infected RBCs having higher expression. There was no association among complement-fixing antibodies, complement activation, and CRP loss. Our findings demonstrate that CRP loss is a pan-species, age-independent mechanism of malarial anemia. Higher levels of CRP and CD47 expression on infected RBCs suggest that parasites are protected from complement-mediated destruction and macrophage clearance. Lack of associations between protective antibodies and CRP loss highlight that complement pathogenic and protective pathways are distinct mechanisms during infection.

## Abstract 2

Loss of complement regulatory proteins (CRPs) on uninfected RBCs is a pan-species and age-independent driver of malaria anemia.

## Introduction

Malaria continues to cause major morbidity and mortality globally; it is responsible for approximately 216 million clinical cases and 445,000 deaths annually (1). Clinical malaria cases are predominantly caused by *Plasmodium falciparum* and *Plasmodium vivax* infection. Anemia is a common clinical manifestation of malaria and a major cause of morbidity and mortality among young children and pregnant women (2, 3). The pathogenic mechanisms of malarial anemia are complex, multifactorial, and not fully defined, particularly for vivax malaria (4, 5). Malarial anemia is not simply due to parasite destruction of infected RBCs but rather results predominately from the loss of uninfected RBCs. For each loss of 1 infected RBC, it is estimated that an additional 8 uninfected RBCs are removed in *P*. *falciparum* (2, 6) and 34 in *P*. *vivax* infection (7). Despite this, the mechanisms underlying this specific loss of uninfected RBCs are poorly understood.

Complement activation is a key mechanism contributing to the loss of RBCs during *P*. *falciparum* malaria anemia (8–10). Complement activation occurs via the classical (antibody binding to C1q), lectin (mannose-binding lectin binding to pathogenic cells), and alternative (basal cleavage of C3 on cell surfaces) cascades (11). Under normal physiological conditions, complement activation on RBCs occurs via basal cleavage of C3 and via collision of RBCs with immune complexes bound to C3b. This process is regulated by complement regulatory proteins (CRPs) on the RBC surface, including complement receptor 1 (CR1/CD35), decay-accelerating factor (DAF/CD55), and protectin (CD59) (12, 13). Alongside complement regulation, CR1 is also an important receptor mediating the clearance of immune complexes from the circulation, whereby C3b-opsonized immune complexes bind to CR1 on RBCs and are taken up by macrophages in the liver and spleen (8). However, this process simultaneously strips CR1 from the RBCs (8), and CD55 is also removed through a similar process (14). The clearance of CRPs reduces the RBC’s capacity to regulate complement deposition (15, 16), thus making RBCs susceptible to complement-mediated destruction through macrophage phagocytosis (13). Erythrophagocytosis by macrophages is also a complement-dependent process during *Plasmodium* infection (14, 17). It is proposed that this process is also regulated by CD47, which is a self-marker expressed on RBC surface (18). During *Plasmodium* infection, proteins released during the rupture of mature blood-stage parasites form immune complexes with antibodies and cause systemic complement activation. This, in turn, results in increased complement deposition on RBCs, removal of RBC CRPs, and anemia (19, 20). It is unknown whether parasite-infected RBCs have developed strategies to avoid this clearance pathway. However, in mice, the *Plasmodium* parasite is shown to preferentially infect RBCs with high CD47 expression to avoid phagocytic clearance (21), suggesting an interplay between parasite invasion and immune clearance mechanisms. Additionally, it is unclear if there are differences in evasion mechanisms of infected RBCs between *P*. *falciparum* and *P*. *vivax*, since the former infects mature RBCs while the latter infects reticulocytes.

The importance of RBC CRP loss and complement deposition as drivers of malaria anemia is supported by studies in African children with *P*. *falciparum* malaria. These studies show that CR1 and CD55 levels are lower on RBCs from children with severe malaria anemia compared with nonanemic children and children with uncomplicated malaria (15, 16, 22–24). Higher levels of C3b deposition on RBCs were observed in individuals with lower CR1 expression, thus increasing their susceptibility to macrophage clearance (14, 16). However, the importance of CRP loss in malaria anemia in adults is unclear (25). Given that CRP expression is age dependent and increases in adults (22), the contribution of this mechanism to malarial anemia in adults may differ from that in children. Further, the role of RBC CRP loss in *P*. *vivax* severe anemia has not been investigated.

Complement activation also has an essential role in antibody-mediated protection against malaria (26). The majority of antibodies that block RBC invasion by *P*. *falciparum* merozoites require complement to function and interact with the first component of the classical pathway, C1q, to activate complement on the parasite surface (26). The possible interplay between the role of systemic complement activation in driving anemia and complement activation required for the function of protective antibodies has not been addressed. Here, we investigated the expression of CRPs and CD47, distinguishing uninfected and infected RBCs, in patients with *P*. *falciparum* and *P*. *vivax* infection with different degrees of anemia from a cohort in Papua, Indonesia. We measured plasma complement activation and complement-fixing antibodies during infection and assessed the relationships of these with CRP loss and anemia. Our data provide important insights into the complex roles of complement in malaria pathogenesis, protection, and parasite invasion mechanism during *P*. *falciparum* and *P*. *vivax* malaria.

## Results

### Study populations.

Expression of CRPs on uninfected and infected RBCs, plasma complement activation, and complement-fixing antibodies were assessed in 121 patients, 79 with *P*. *falciparum* and 42 with *P*. *vivax* malaria. There were no differences in age, sex, and parasitemia between anemia groups (Table 1).

### CR1 and CD55 on uninfected RBCs are reduced in P.

**Figure 1:**
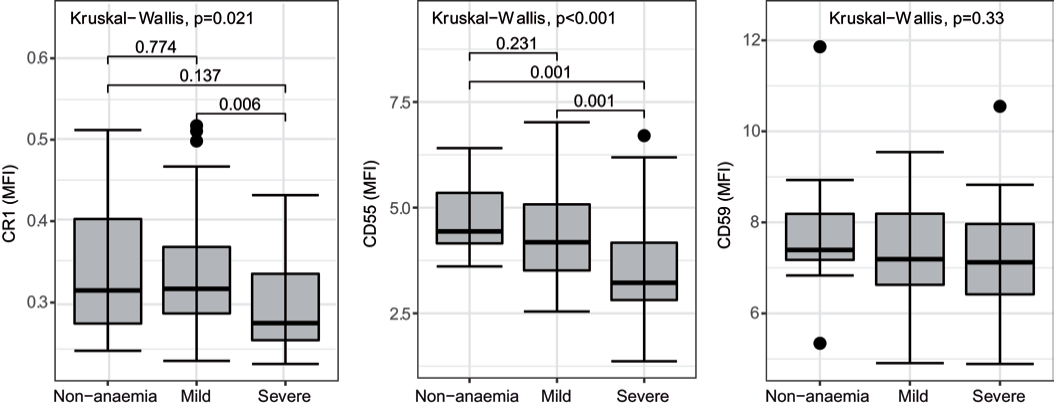
Level of CRPs on uninfected RBC surfaces from *P.* ***falciparum*–infected patients.** Expression of CR1, CD55, and CD59 compared among 3 different anemia statuses (nonanemia, *n* = 11; mild anemia, *n* = 38; severe anemia, *n* = 30). Kruskal-Wallis test and Mann-Whitney nonparametric test between groups is indicated. Lower and upper hinges represent first and third quartiles, and whisker lines correspond to highest and lowest values no further than the 1.5 interquartile range from the hinges. Data beyond the whisker lines are treated as outliers. Median line is indicated across the box. CRPs, complement regulatory proteins.

*falciparum malaria with severe anemia*. We first assessed CRPs and CD47 expression on uninfected RBCs from patients infected with *P*. *falciparum* (Supplemental Figure 1; supplemental material available online with this article; https://doi.org/10.1172/jci.insight.124854DS1). Expression of RBC CR1 and CD55 differed significantly among patients with different degrees of anemia (Figure 1). The lowest expression of CR1 and CD55 was seen in patients with severe anemia. There was no difference in CD59 on RBCs among anemia categories (Figure 1). Consistent with these findings, CR1 and CD55, but not CD59, were correlated positively with hemoglobin (Hb) concentration (g/dl) (Supplemental Figure 2; Spearman’s, CR1, r = 0.29, *P* = 0.011; CD55, r = 0.45, *P* < 0.001; CD59, r = 0.2, *P* = 0.086). No differences in CD47 expression were observed across anemia categories (Supplemental Figure 3).

### CR1 on uninfected RBCs is reduced in anemic patients with P.

**Figure 2:**
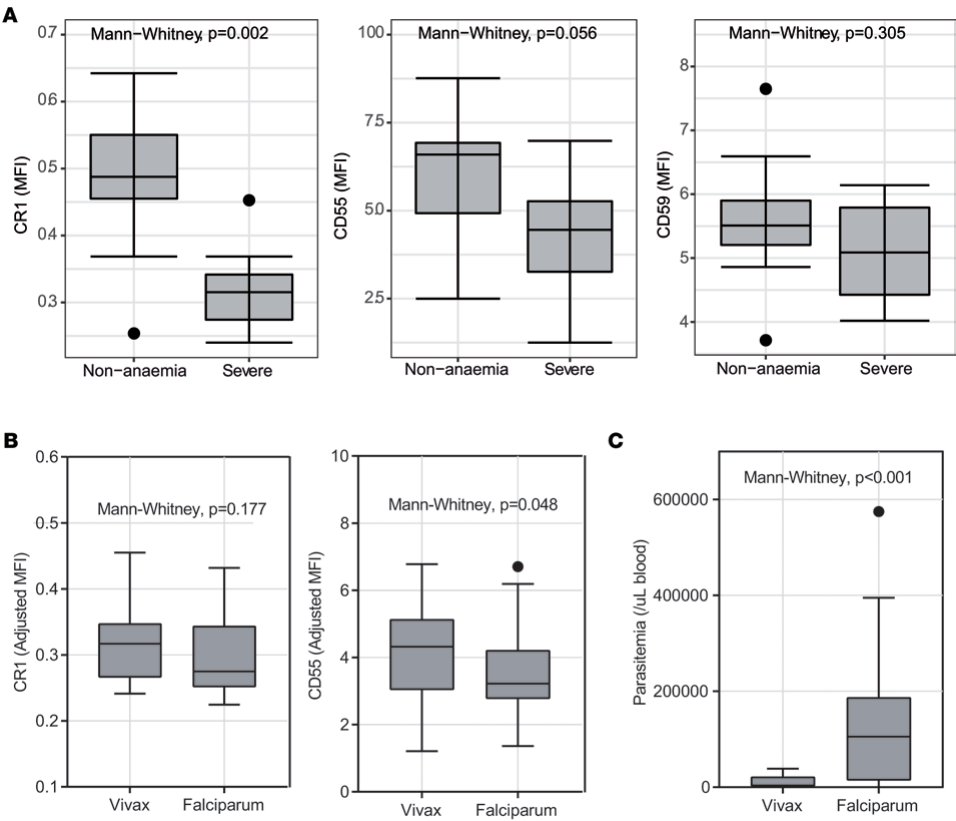
Levels of CRPs on uninfected RBCs surfaces from *P*. ***vivax*–infected patients in comparison with*P*.*falciparum*–infected patients.** (**A**) Level of complement regulatory proteins (CRPs) on uninfected RBC surfaces from *P*. *vivax*–infected patients. Expression of CR1, CD55, and CD59 was compared between 3 among anemia statuses (nonanemia, *n* = 10; severe anemia, *n* = 12). (**B**) Comparison of CR1 and CD55 expression on uninfected RBCs between *P*. *vivax* (*n* = 12) and *P*. *falciparum* (*n* = 30) patients with severe anemia. (**C**) Comparison of parasite biomass (parasite/μl of blood) between *P*. *vivax* (*n* = 13) and *P*. *falciparum* (*n* = 30) patients with severe anemia. Mann-Whitney nonparametric test between groups is indicated. Lower and upper hinges represent first and third quartiles, and whisker lines correspond to highest and lowest values no further than 1.5 interquartile range from the hinges. Data beyond the whisker lines are treated as outliers. Median line is indicated across the box.

*vivax malaria*. The expression of CRPs and CD47 was assessed on uninfected RBCs from patients with *P*. *vivax* infection. Due to limitations in sample availability, only nonanemic and severely anemic patients were tested. Uninfected RBCs from patients with severe malaria anemia had lower surface expression of CR1 than those from patients with nonanemic infection (Figure 2A). Lower levels of CD55 were also observed in severely anemic patients, although this difference was not statistically significant (Figure 2A). There was no difference in CR1 or CD55 expression between adults and children (Supplemental Figure 4) and no difference in CD59 expression on RBCs between nonanemic and severely anemic patients (Figure 2A). As for *P*. *falciparum*, there was also no difference in CD47 expression between nonanemic and anemic patients (Supplemental Figure 3). In patients with severe anemia, the expression of CR1 on uninfected RBCs between *P*. *vivax* and *P*. *falciparum* was similar; however, the expression of CD55 in *P*. *falciparum* malaria was significantly lower than that in *P*. *vivax* malaria (Figure 2B). Concurrently, parasitemia was significantly lower in *P*. *vivax*–infected patients compared with *P*. *falciparum*–infected patients (Figure 2C).

To assess whether changes to CRP levels were restricted to mature RBCs, we also assessed the frequency and CRP expression of reticulocytes in *P*. *vivax–*infected patients. The percentage of reticulocytes was significantly higher in severely anemic than nonanemic *P*. *vivax*–infected patients (Supplemental Figure 5A). Overall, expression of CR1, CD55, and CD59 was significantly higher in reticulocytes when compared with uninfected mature RBCs (Supplemental Figure 6); however, reticulocyte CR1 levels were significantly lower in severely anemic than nonanemic patients (Supplemental Figure 5B), indicating that CRP loss was not restricted to mature RBCs.

### P.

**Figure 3:**
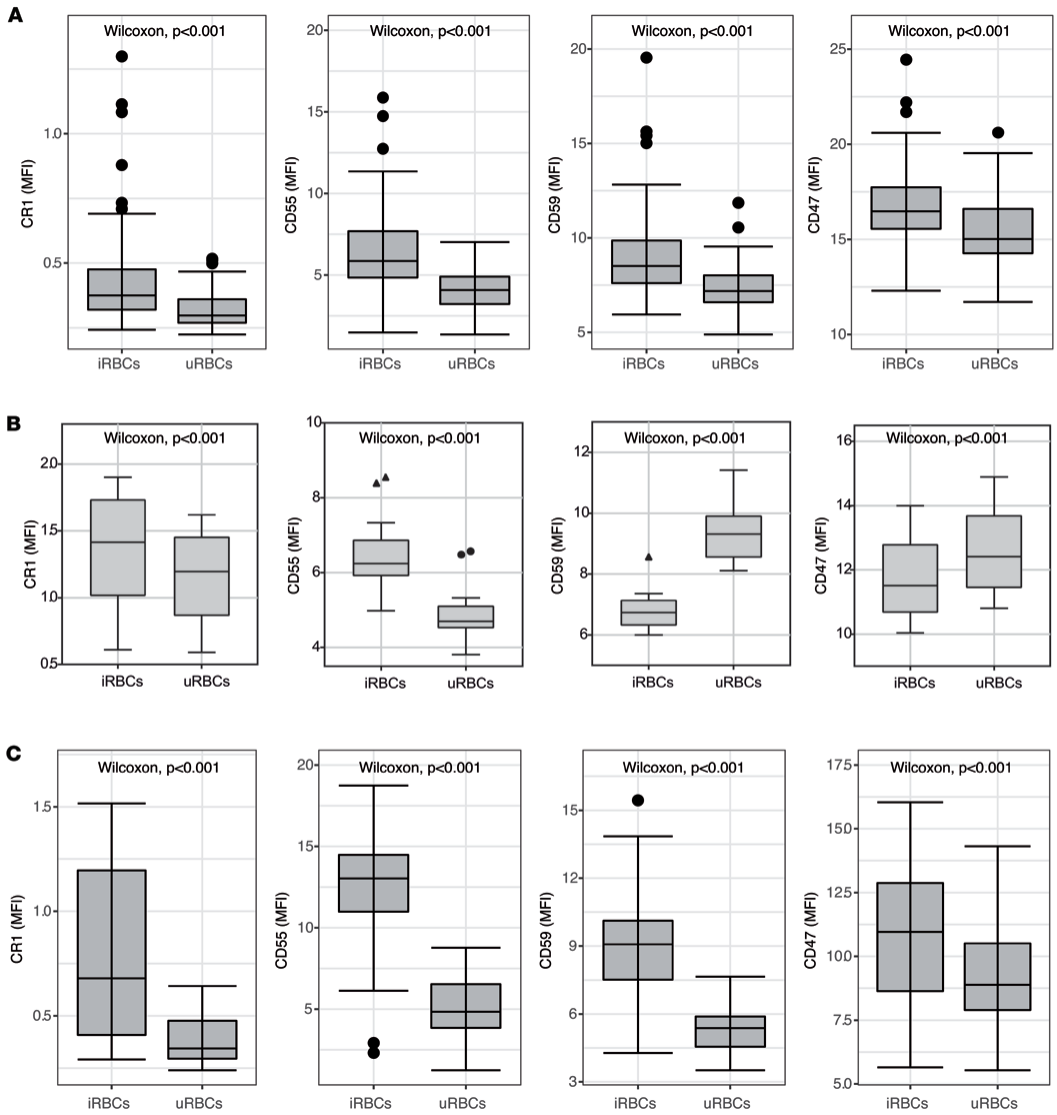
Comparison of CRPs and CD47 expression between uninfected and infected RBCs from *P.* ***falciparum*and*P. vivax*malaria.** (**A**) Expression of CR1, CD55, CD59, and CD47 was compared between uninfected RBCs and infected RBCs (SYBR Green positive) from *P*. *falciparum* malaria (CR1, *n* = 70; CD55, *n* = 70; CD59, *n* = 69; CD47, *n* = 69). Wilcoxon signed-ranked test is indicated. (**B**) Expression of CR1, CD55, CD59, and CD47 was compared using the in vitro *P*. *falciparum* D10-GFP model on RBCs from healthy naive volunteers (*n* = 8, tested in duplicates). iRBCs, infected RBCs; uRBCs, uninfected RBCs. (**C**) Expression of CR1, CD55, CD59, and CD47 was compared between uninfected RBCs and infected RBCs (SYBR Green positive) from *P*. *vivax* malaria (CR1, *n* = 16; CD55, *n* = 16; CD59, *n* = 16; CD47, *n* = 16). Wilcoxon signed-ranked test is indicated. Lower and upper hinges represent first and third quartiles, and whisker lines correspond to highest and lowest values no further than 1.5 interquartile range from the hinges. Data beyond the whisker lines are treated as outliers. Median line is indicated across the box. CRPs, complement regulatory proteins.

*falciparum– and P*. *vivax–infected RBCs have higher expression of CRPs and CD47*. We next compared the level of CRPs between uninfected and infected RBCs within individual patient samples. CR1, CD55, and CD59 were significantly higher in *P*. *falciparum*–infected RBCs compared with uninfected RBCs within the same patient (Figure 3A). Similarly, CD47 expression was significantly higher in infected compared with uninfected RBCs (Figure 3A). To investigate whether the higher CRP levels on infected RBCs in patient samples were due to preferential invasion of RBCs with high expression of CR1 and CD55, we assessed the concentration of CRPs and CD47 on newly invaded *P*. *falciparum*–infected RBCs in vitro. Consistent with the ex vivo CRP expression from patients, infected RBCs in culture had higher expression of CR1 and CD55 than uninfected RBCs (Figure 3B). In contrast to the ex vivo results, CD59 and CD47 expression was lower on infected RBCs compared with uninfected RBCs following invasion in vitro. Expression of CRPs and CD47 was also higher in *P*. *vivax–*infected RBCs compared with uninfected RBCs within the same patient (Figure 3C).

### CRP expression on RBCs is not associated with plasma complement activation or complement-fixing antibodies.

To investigate the potential overlap in CRP loss, complement activation, and functionally protective complement-fixing antibodies, we measured levels of C1q-fixing antibodies against *P*. *falciparum* merozoites or *P*. *vivax* merozoite antigen PvMSP3α (block I–II), along with levels of complement activation (*n* = 76 and 24, respectively), within our patient cohort. There was no association between levels of C1q-fixing antibodies and CRP RBC expression for either *P*. *falciparum*– or *P*. *vivax*–infected malaria patients (Table 2 and Supplemental Figures 7 and 8) and no association between levels of C1q-fixing antibodies and serum complement activation (as indicated by quantification of complement activation products C3a and C5a) in either *P*. *falciparum* or *P*. *vivax* malaria (Table 2). There was no association between Hb level and complement activation (assessed by C3a and C5a levels) in either *P*. *falciparum–infected* (Spearman’s, C3a, *P* = 0.60; C5a, *P* = 0.71) or *P*. *vivax*–infected (Spearman’s: C3a, *P* = 0.18; C5a, *P* = 0.19) patients (Supplemental Figure 9). Taken together, our data show no link between functional protective complement-fixing antibodies and activation of complement or loss of CRPs on RBCs during *P*. *falciparum* or *P*. *vivax* malaria.

## Discussion

Here, we show that significant loss of CRPs on RBCs occurs during both *P*. *falciparum* and *P*. *vivax* anemia. Our findings also demonstrate that loss of CRPs is not confined to childhood malaria (22), demonstrating CRP loss as a pan-species, age-independent mechanism of anemia. Of note, RBC CRP reduction was restricted to uninfected RBCs, with infected RBCs having relatively higher CRP expression, suggesting that parasite invasion pathways also protect parasites from immune clearance mechanisms. We show that *P*. *falciparum* parasites preferentially invade RBCs with high CR1 and CD55 in vitro, consistent with the use of these proteins as invasion ligands (27, 28) and highlighting the use of these invasion pathways not only for parasite growth, but also as immune evasion strategies. Importantly, the loss of CRPs in malaria anemic patients was not associated with functional complement-fixing antibodies or generalized activation of serum complement. Overall, our data highlight the multifaceted role of complement factors in pathogenesis, functional protective mechanism, and parasite replication.

The reduced expression of CR1 and CD55 on RBCs in severe anemia from both *P*. *falciparum* or *P*. *vivax* supports the hypothesis that RBCs with lower expression of CRPs are associated with RBC destruction and that this is a species-transcending mechanism of malaria anemia. Previous studies have demonstrated that the loss of CRPs is associated with increased C3b deposition on RBC (16), resulting increased susceptibility to erythrophagocytosis by macrophage (17). While well recognized as a complication of *P*. *falciparum* infection, anemia is also an important cause of morbidity and mortality from *P*. *vivax* malaria (29, 30), but mechanisms of anemia in *P*. *vivax* are poorly understood (31). *P*. *vivax* infection is known to cause greater destruction of RBCs per parasite than *P*. *falciparum* (6, 7). Consistent with these data, our study demonstrates that, in patients with severe anemia, CR1 levels on uninfected RBCs were comparable between *P*. *vivax–* and *P*. *falciparum*–infected patients, despite significantly lower parasite biomass in *P*. *vivax–*infected patients. Although the precise mechanism by which vivax malaria evoked greater CR1 reduction than falciparum is not understood, higher inflammatory responses seen in *P*. *vivax* compared with *P*. *falciparum* infection could be a contributing factor (5, 32, 33). Additionally our study is the first to our knowledge to report CRP loss in malaria anemia in non-African and adult populations. CRP expression on RBCs is age dependent and increases with age, and lower expression of CRPs in young children is thought to exacerbate the risk of severe anemia in infants (22). Despite age-dependent CRP expression, we found that CRP loss is also a likely mechanism driving anemia in adults, who at our study site remain at risk of malaria due to lower transmission intensity

In this study, we distinguished infected RBCs from the uninfected in malaria patients. The loss of uninfected RBCs in both *P*. *falciparum* and *P*. *vivax* malaria is well characterized and contributes significantly more to malarial anemia than the loss of infected RBCs (5, 31). Despite this, the mechanisms underlying the specific loss of uninfected RBCs are obscure. Our study highlights that the loss of CRPs in both species of malaria is particularly apparent in uninfected RBCs, whereas infected RBCs of both *P*. *falciparum* and *P*. *vivax* malaria have higher expression of CR1, CD55, and CD59 than uninfected RBCs. Further, our in vitro data assessing *P*. *falciparum* invasion of RBCs confirm that CR1 and CD55 are higher in parasitized compared with uninfected RBCs, consistent with both being important RBC receptors that facilitate *P*. *falciparum* invasion (27, 28). It is currently unknown if CD59 or CD47 are used by *P*. *falciparum* as invasion receptors or whether *P*. *vivax* uses CR1 or CD55 as receptors for RBC invasion. However, transferrin receptor 1 (TfR1/CD71) is expressed on reticulocytes and has been identified as a key invasion ligand for *P*. *vivax* (34). Our data show that reticulocytes and immature RBCs also express high levels of CR1, CD55, CD59, and CD47, consistent with previous studies (35–37). High levels of CR1 and CD55 may prevent complement attack on infected RBCs. Additional high CD59 expression may mediate protection from complement-mediated lysis (38). Similarly, mouse models have shown that *Plasmodium* parasites preferentially invade CD47 expressing RBCs in order to escape macrophage clearance (21). Thus, the dependency of invasion via CD71 results in invasion into RBCs with high CRPs and CD47. However, we also saw some reduction of reticulocyte CRP expression in severely anemic patients, indicating that CRP loss is not restricted to mature RBCs. Thus, our data suggest that parasites preferentially infect high CRP-expressing RBCs to mediate invasion as well as immune evasion to avoid parasite clearance.

Serum complement also plays an important role in the function of protective *P*. *falciparum* antibodies (26, 39). Increased IgG deposition on RBCs was previously linked to CRP loss and anemia (23); however, it is unknown if protective complement-fixing antibodies play a role in this pathogenic pathway. Importantly, within this study, we saw no association between complement-fixing antiparasite antibodies and CRP RBC levels or sera complement activation, suggesting that protection mechanisms of complement fixation by antibodies are not linked with malaria anemia pathogenesis. Our findings have important implications for the development of vaccines that aim to induce complement-fixing antibodies, as it suggests that such antibody induction will not increase the risk of anemia mediated by complement activation. The lack of association between complement activation in pathogenesis and disease may reflect differences in the subclasses of antibodies that are implicated in each mechanism. The IgG subclasses that fix complement to mediate protection are mainly IgG1 and IgG3 (26). This IgG1 and IgG3 dominance to *P*. *falciparum* and *P*. *vivax* antigens was also previously demonstrated within the current study population (40). However, immune complexes formed with IgG1 and IgG3 bind poorly to CR1 (41), suggesting that these subclass protective responses may not contribute to CRP loss on RBCs. Indeed, one study in western Kenya reported that severe malarial anemia is associated with high levels of IgG4-containing immune complexes (42), suggesting that nonprotective IgG subclasses are the drivers of CRP removal and anemia. While complement activity was previously linked with severe malarial anemia in children (9, 43), we observed no association between total complement activation (assessed by C3a and C5a levels) and anemia in our populations. Discrepancies between our findings and previous reports (9, 43) may be due to differences in populations or in experimental methods used to assess complement activation and complement activation products (9) or complement factors (43).

In conclusion, we demonstrate that reduced expression of CRPs on the surface of uninfected RBCs likely plays a critical role in the hitherto unexplained loss of uninfected RBCs in both *P*. *falciparum* and *P*. *vivax* malaria anemia. CRP expression was higher on infected compared with uninfected RBCs, suggesting that parasite invasion of RBCs via CRP receptor pathways may additionally act as an immune evasion strategy to protect parasites from complement-mediated clearance. Furthermore, CRP loss is not linked to complement-fixing antibodies associated with protection. Our data provide important insights into the complex roles of complement in malaria pathogenesis and between protection and parasite invasion mechanisms during *P*. *falciparum* and *P*. *vivax* malaria.

## Methods

### Study cohorts.

Patients with malaria were enrolled between 2004 and 2007 in Timika, on the southern province of Papua, Indonesia. *P*. *falciparum*–infected adults were ≥18-year-old patients with acute moderately severe or severe malaria, as described previously (44, 45). Exclusion criteria included pregnancy or breastfeeding, patients treated with antimalarials for >18 hours, mixed *P*. *falciparum* and *P*. *vivax* infections, and Hb <60 g/l. Patients were treated with intravenous quinine or artesunate in accordance with prevailing national policy guidelines. *P*. *vivax* samples were collected from patients with acute vivax malaria enrolled in a clinical trial of artemisinin combination therapy (46) and in vitro efficacy studies (47). Pregnant women and children under 10 kg were excluded.

Participants in the current study were selected based on the availability of glycerol-preserved RBCs and plasma samples with a range of Hb concentrations. Selected participants were grouped into categories, nonanemia (NA), mild anemia (MA), and severe anemia (SA), based on World Health Organization anemia categories (48). Mild/moderate anemia was defined as an Hb concentration between 8.0 and 12.9 g/dl for men aged 15 years and older, 8.0–11.9 g/dl for women aged 15 years and older, and 8.0–11.4 g/dl for children younger than 15 years. Severe anemia was defined as a Hb concentration below 8.0 g/dl in all age groups. Plasma samples were available from 76 patients with *P*. *falciparum* and 42 patients with *P*. *vivax* infection. Glycerol-preserved RBCs were available for 79 individuals with *P*. *falciparum* and 22 individuals with *P*. *vivax*, which were limited to the severe and nonanemia categories.

### Parasite culture and synchronization, merozoite isolation, and growth assay.

Both the *P*. *falciparum* D10-GFP and 3D7 strain were cultured and synchronized as described previously (49, 50). Briefly, RPMI-HEPES containing 10% of a 50:50 ratio of human serum and Albumax I (Gibco) was used as culture medium. D10-GFP parasites were synchronized using 30 IU heparin solution (Pfizer) at a final concentration of 20 IU/ml (49).

Schizont-stage (40–46 hours after invasion) parasites were purified from uninfected RBCs using the VarioMACS magnet system and CS column (Miltenyi Biotec) (49). Purified schizont-stage parasites were incubated with E64 at 10 μM (MilliporeSigma) for 6–8 hours, and the parasites were filtered and pelleted through a 1.2-μm filter unit (Sartorius) to isolate merozoites. Isolated merozoites were used for ELISA assay.

To measure expression of CRPs in parasite culture following invasion, purified schizont-stage D10-GFP parasites were added to RBCs from healthy and malaria-naive individuals (*n* = 8) and incubated for 24 hours. Another set of matched RBCs without schizonts were used as controls. After 24 hours, the culture samples were stained and analyzed by flow cytometry.

### Flow cytometric measurement of CR1, CD55, CD59, and CD47 on RBC surface.

Frozen glycerol-preserved RBC samples from patient cohorts were used to determine the expression of CR1, CD55, CD59, and CD47 as previously described (15, 51). Control RBC samples were obtained from healthy malaria-naive individuals and cryopreserved (*n* = 6). Antibody staining of CRPs was done individually with the addition of SYBR Green (Thermo Fisher Scientific) for parasite staining, and staining condition was optimized to maximize fluorescent intensity (Supplemental Figure 1). Briefly, a 5-μl erythrocyte pellet was added into a well of 96-well V-bottom plate (Greiner). Twenty-five microliters of SYBR Green (diluted 1:10,000 in DMSO) and individual anti-CRP antibodies were added to a total volume of 50 μl with PBS. The following antibodies were used to stain the red cells: mouse anti-human CR1 BV421 (clone E11, Becton Dickinson), CD55 APC (clone IA10, Becton Dickinson), CD59 BV421 (clone p282 H19, Becton Dickinson), CD47 BV421 (clone B6H12, Becton Dickinson), and isotype controls for each (Becton Dickinson). Samples were stained for 25 minutes in the dark at room temperature. Samples were washed 3 times with PBS before acquisition on a FACS Gallios (Beckman Coulter) at 100,000–500,000 events per sample, and data were analyzed with Kaluza 1.3 (Beckman Coulter). For median fluorescent intensity (MFI) measurements of infected RBCs, a 0.1% parasitemia was used as a cutoff point. Mouse anti-human CD71 PE-Cy7 (clone CY1G4, Biolegend) antibody was added in *P*. *vivax* sample staining to the exclude CD71^+^ (immature reticulocytes) population (Supplemental Figure 1).

To compare CRPs and CD47 expression between *P*. *falciparum* and *P*. *vivax* samples, malaria sample MFI was normalized to control MFI and converted into arbitrary units.

### Complement deposition assay with ELISA.

ELISA procedure was carried out as described previously (26, 52). Ninety-six flat-bottom Maxisorp plates (Nunc) were coated overnight at 4°C with purified *P*. *falciparum* merozoites. Purified merozoites were isolated from 50 ml parasite culture (3% hematocrit, ~3% parasitemia) that would normally yield 2 × 10^8^ merozoites (49). Washing steps were done 3 times with PBS-Tween (0.5% w/v). The plates were then blocked with 1% casein for 2 hours at 37°C. Plasma samples (1:250 dilution) from *P*. *falciparum*–infected patients were added to the plates and incubated for 2 hours at room temperature. Recombinant C1q (10 μg/ml, Quidel) was added and C1q was detected with 1:2,000 rabbit anti-C1q antibodies (Dako) and 1:4,000 goat anti-rabbit HRP (Bio-Rad). Tetramethylbenzidine (MilliporeSigma) was added as a substrate, and the reaction was stopped with 1 M HCl. Absorbance of the reaction was read at 450 nm.

For *P*. *vivax* patient samples, the plates were coated with recombinant PvMSP3α block I–II region (nucleotides 316–2,058) protein. The recombinants proteins were amplified from *P*. *vivax* (Belem strain), expressed with vector containing a C-terminal 6× His Tag, and purified as described previously (53). Subsequent steps were similar to those described above.

### Complement activation assessed by C3a and C5a ELISA.

Plasma samples from *P*. *falciparum*–infected (NA, *n* = 5; MA, *n* = 11; SA, *n* = 8) and *P*. *vivax*–infected (NA, *n* = 8; MA, *n* = 11; SA, *n* = 8) patients were available for complement activity assay. Concentration of complement activation products (C3a and C5a) was measured using commercial ELISA kits (eBioscience) as per the manufacturer’s instructions.

### Statistics.

All analyses were performed in STATA (version 15.0), RStudio (version 1.0.153), and GraphPad Prism (version 7.03). Differences in CRPs and CD47 expression on uninfected RBCs were compared among anemia groups using Kruskal-Wallis and Mann-Whitney nonparametric test. Correlations between the complement-fixing antibodies and CRPs and between complement activation and Hb level were determined using Spearman’s nonparametric method. For the comparison of CRPs and CD47 between uninfected and infected RBCs, Wilcoxon signed-rank test was used. A *P* value less than 0.05 was considered significant.

### Study approval.

Written informed consent was obtained from all study participants or, in the case of children, parents or guardians. For clinical cohorts, the study was approved by the ethics committees of the Northern Territory Department of Health (Darwin City, Northern Territory, Australia), Menzies School of Health Research, the Indonesian National Institute of Health Research and Development (Jakarta, Indonesia), and the Oxford Tropical Research Committee (Oxford, United Kingdom).

## Author contributions

DAO and MJB designed research study. DAO conducted experiments. DAO, MJB, JGB, NMA, and RNP analyzed data. EK, JRP, NMA, and RNP supervised the clinical studies and sampling. DAO, MJB, and NMA led manuscript preparation with feedback from all authors.

## Supplementary Material

Supplemental data

## Figures and Tables

**Table 2:**
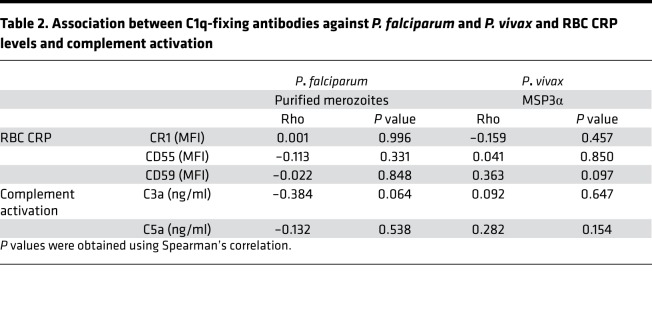
Association between C1q-fixing antibodies against *P. falciparum* and *P. vivax* and RBC CRP levels and complement activation

**Table 1:**
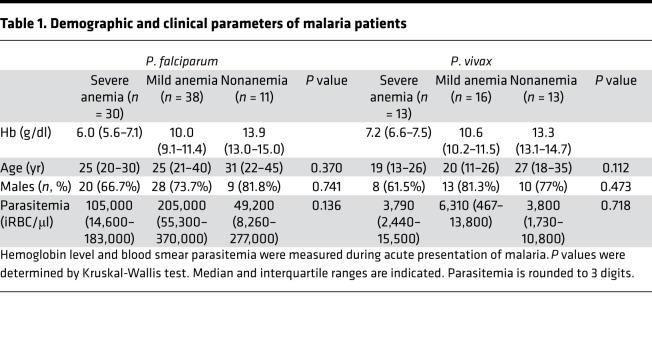
Demographic and clinical parameters of malaria patients

## Version 1 11/15/2018

Electronic publication
