# Supplementary material for: Loss of complement regulatory proteins on uninfected erythrocytes in vivax and falciparum malaria anemia
Source: JCI Insight. 2018 Nov 15;3(22):e124854. doi: 10.1172/jci.insight.124854 (PMC6303009; doi:10.1172/jci.insight.124854)
Supplement: Supplemental data [file jciinsight-3-124854-s117.pdf]

Supplementary Data

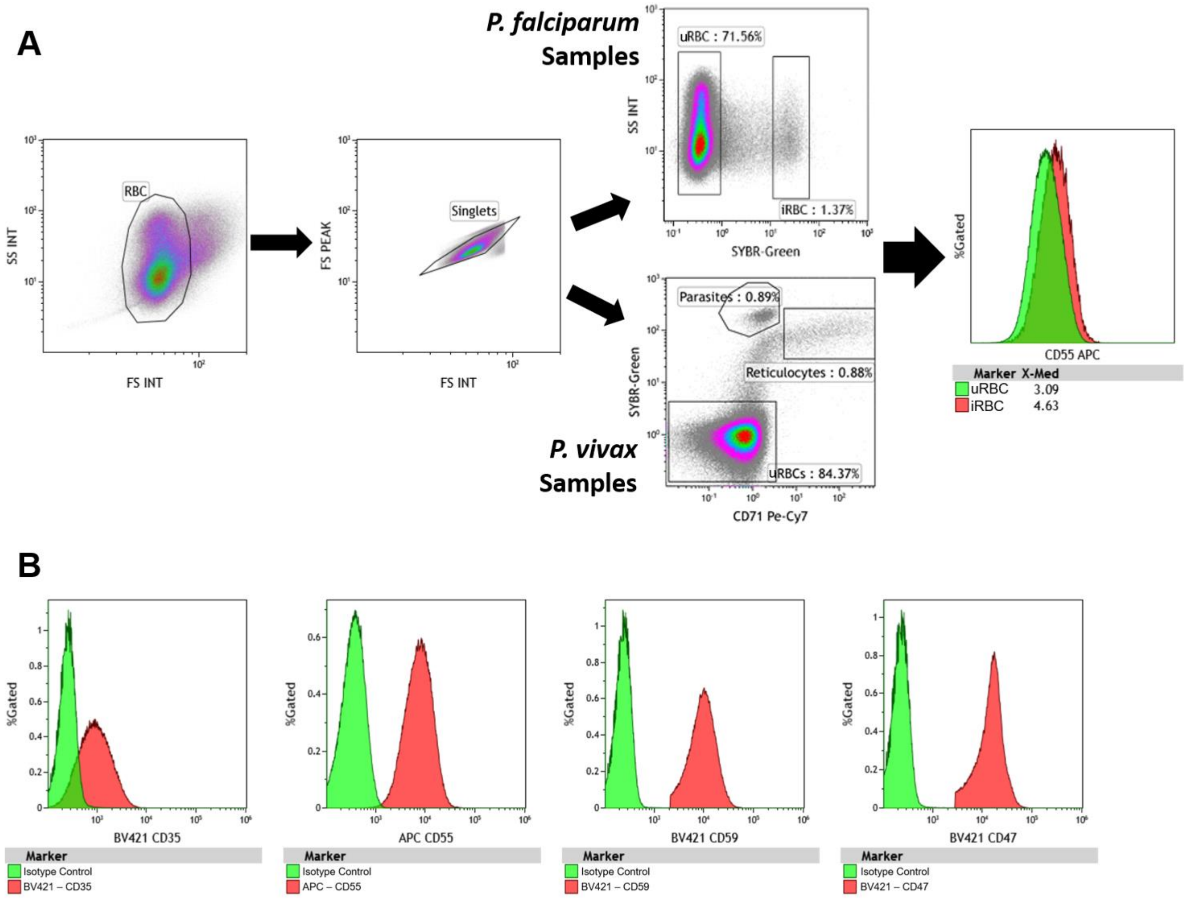

Figure S1. Gating strategy for measuring CRPs on RBCs. (A) RBCs were characterized into uninfected RBC (uRBC) and infected RBC (iRBC) using SYBR-Green DNA dye; uRBC = SYBR-Green (-), iRBC = SYBR-Green (+). For *P. vivax* samples, CD71 Pe-Cy7 was added to distinguished reticulocytes (CD71+). Histogram graph indicates CRP MFI; green = uRBC, red = iRBC. **(B)** CRP and CD47 antibody staining optimisation. Staining volumes were optimised to maximise fluorescent intensity (x-axis) between negative isotype control and antibody stains. Histogram graph indicates MFI; green = isotype control, red = CRP and CD47 antibodies.

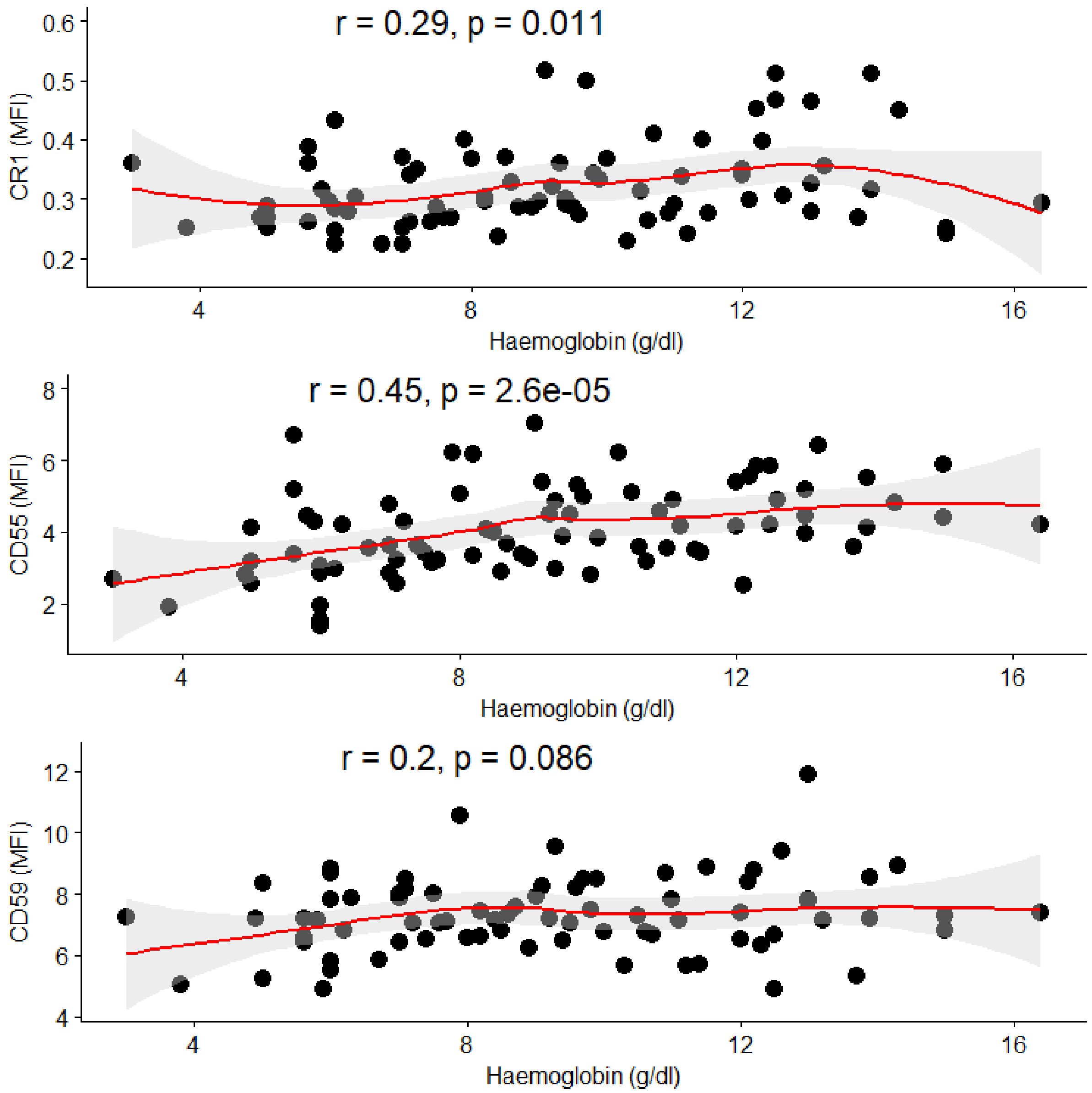

Figure S2. Relationship between CRPs expression and haemoglobin level in *P. falciparum* infected patients. Level of CR1, CD55, and CD59 was correlated with haemoglobin level from patients with *P. falciparum* infection (n = 79). Spearman's correlation coefficient is indicated. Red line indicates Loess non-parametric regression and gray band indicates 95% confidence interval.

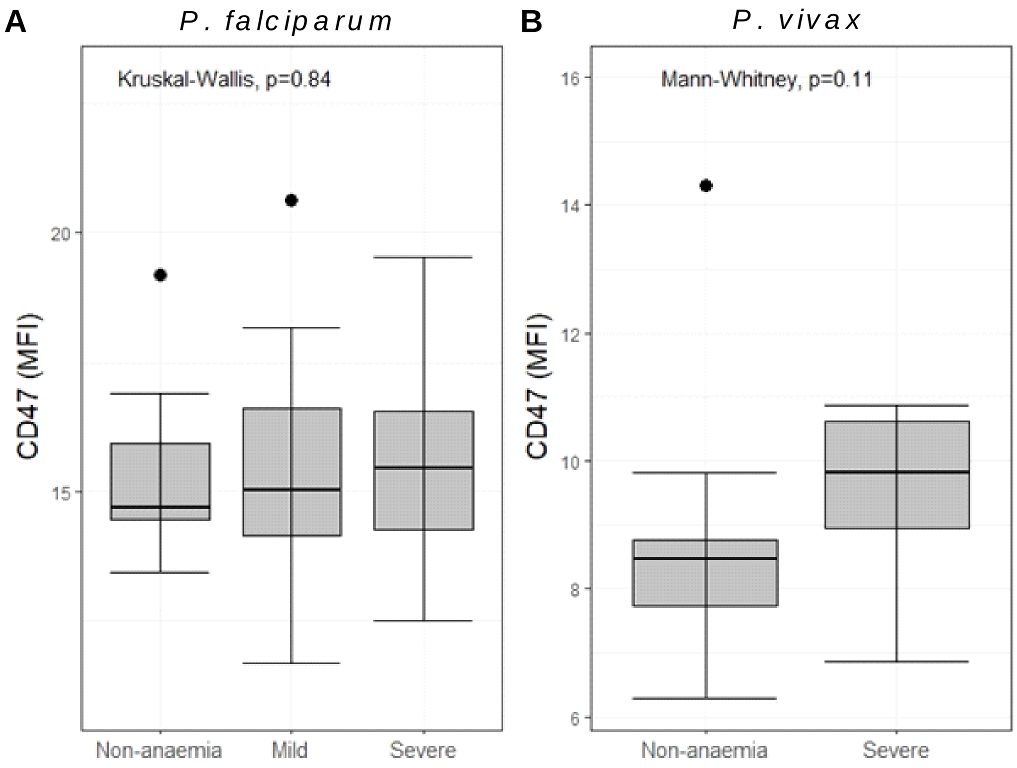

Figure S3. Level of CD47 on uninfected RBC surface. A) Expression of CD47 from *P. falciparum* infected patients was compared between 3 different anaemia statuses (non-anaemia, n = 11; mild-anaemia, n = 37; severe anaemia, n = 30). **B)** Expression of CD47 from *P. vivax* infected patients was compared between non-anaemia (n = 9) and anaemia (n = 10) status. Kruskal-Wallis test and Mann-Whitney non-parametric test between groups is indicated. Lower and upper hinges represent first and third quartiles, and whisker lines correspond to highest and lowest values no further than 1.5 interquartile range from the hinges. Data beyond the whisker lines are treated as outliers. Median line is indicated across the box.

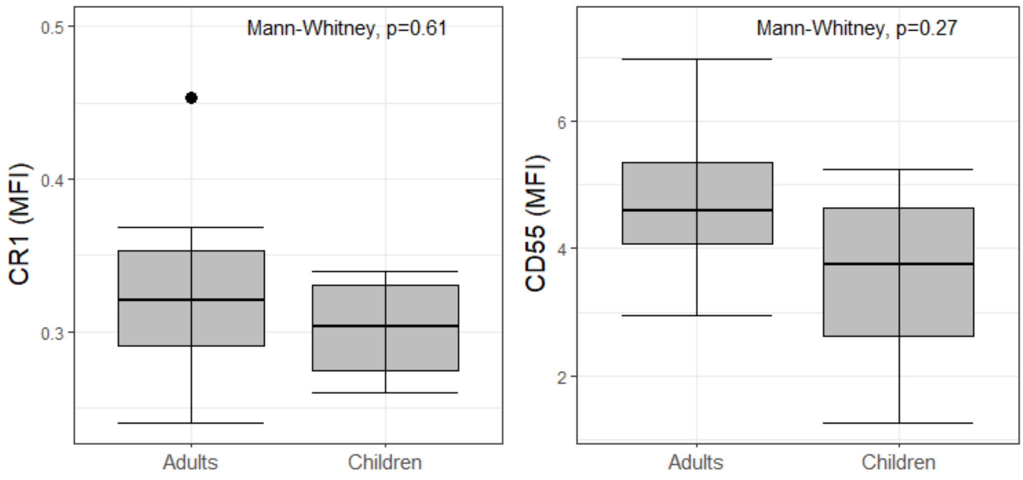

Figure S4. Uninfected RBCs CRPs level comparison between children and adults with *P. vivax* malaria. Expression of CR1 and CD55 was compared between children (n = 4) and adults (n = 8) with severe anaemia. Children <15 years old; adults ≥15 years old. Mann-Whitney non-parametric test between groups is indicated. Lower and upper hinges represent first and third quartiles, and whisker lines correspond to highest and lowest values no further than 1.5 interquartile range from the hinges. Data beyond the whisker lines are treated as outliers. Median line is indicated across the box.

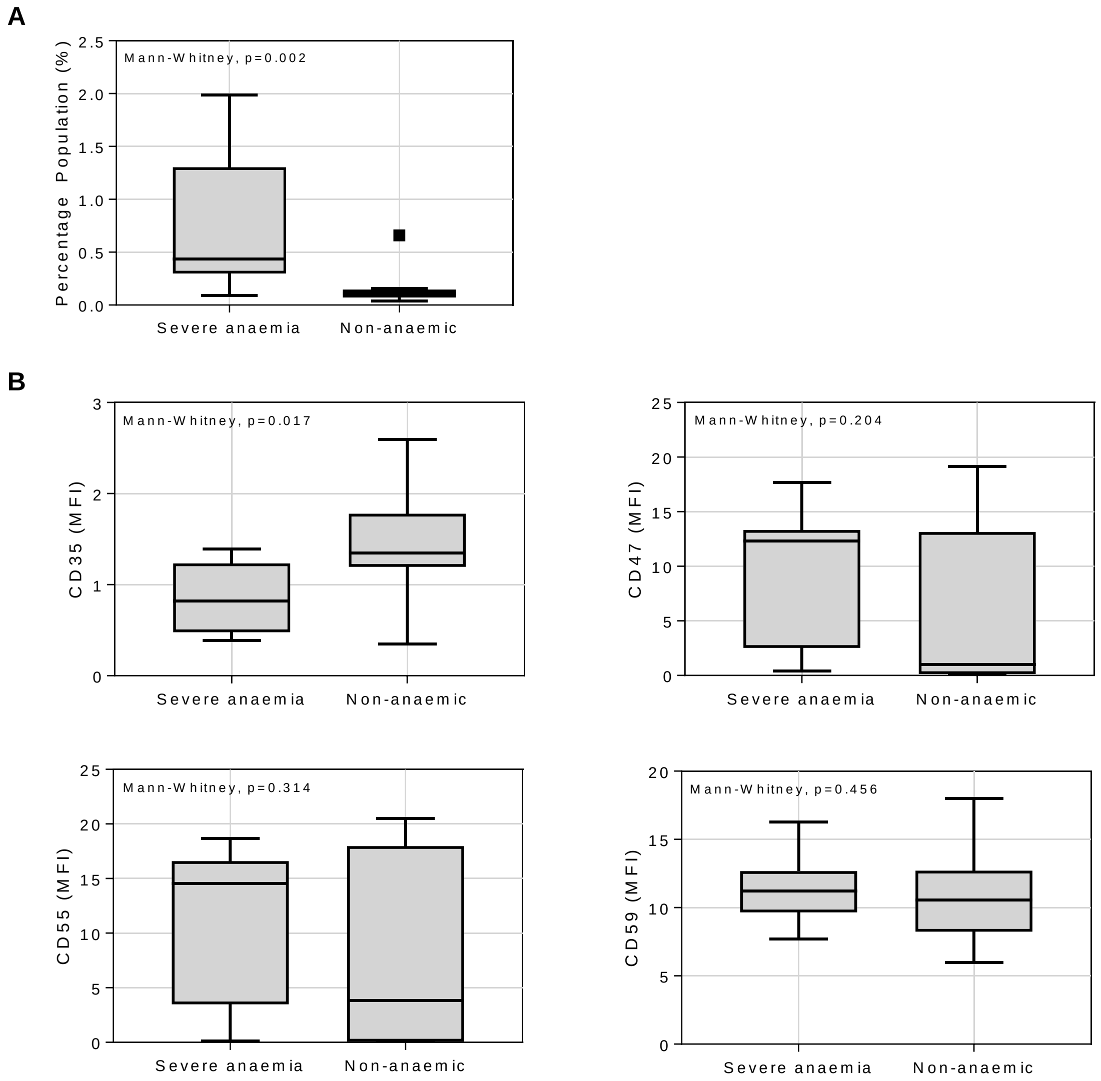

Figure S5. Comparison of reticulocytes population percentage and CRPs and CD47 expression between severe anaemia (n = 12) and non-anaemia (n = 9) *P. vivax* infected patients. A) Percentage of reticulocytes population measured by flow cytometry. **B)** Expression of reticulocytes CR1, CD55, CD59, and CD47. Mann-Whitney non-parametric test between groups is indicated. Lower and upper hinges represent first and third quartiles, and whisker lines correspond to highest and lowest values no further than 1.5 interquartile range from the hinges. Data beyond the whisker lines are treated as outliers. Median line is indicated across the box.

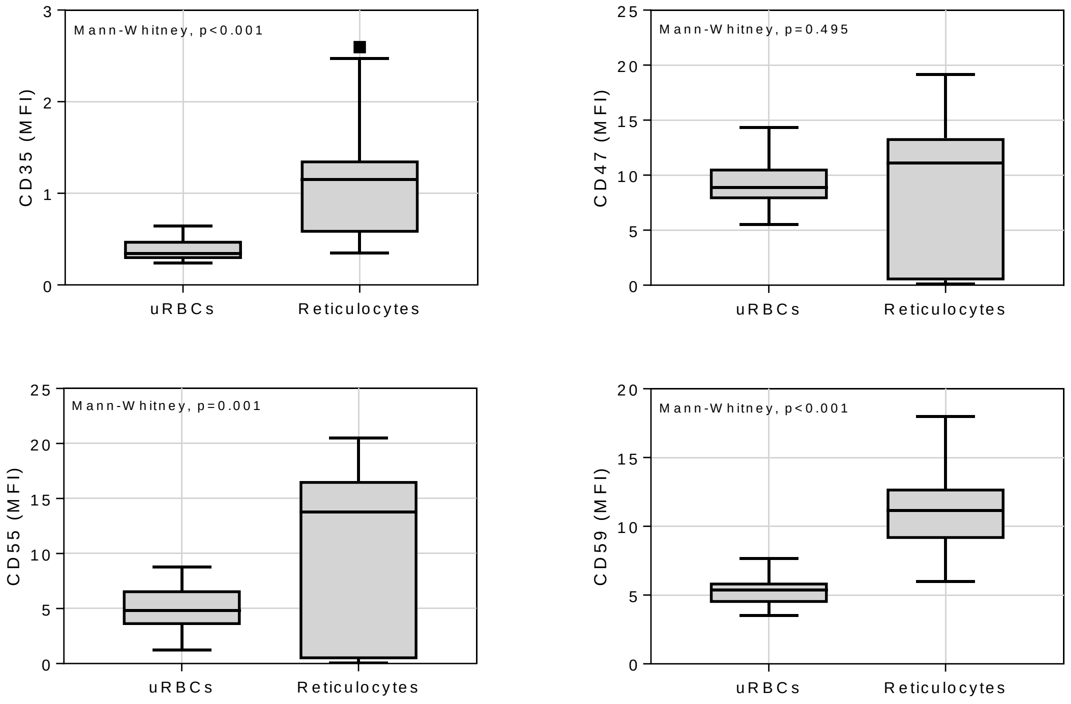

Figure S6. Comparison of CRPs and CD47 expression between uninfected mature RBCs (n = 24) and reticulocytes (n = 24) from *P. vivax* infected patients. Mann-Whitney non-parametric test between groups is indicated. Lower and upper hinges represent first and third quartiles, and whisker lines correspond to highest and lowest values no further than 1.5 interquartile range from the hinges. Data beyond the whisker lines are treated as outliers. Median line is indicated across the box.

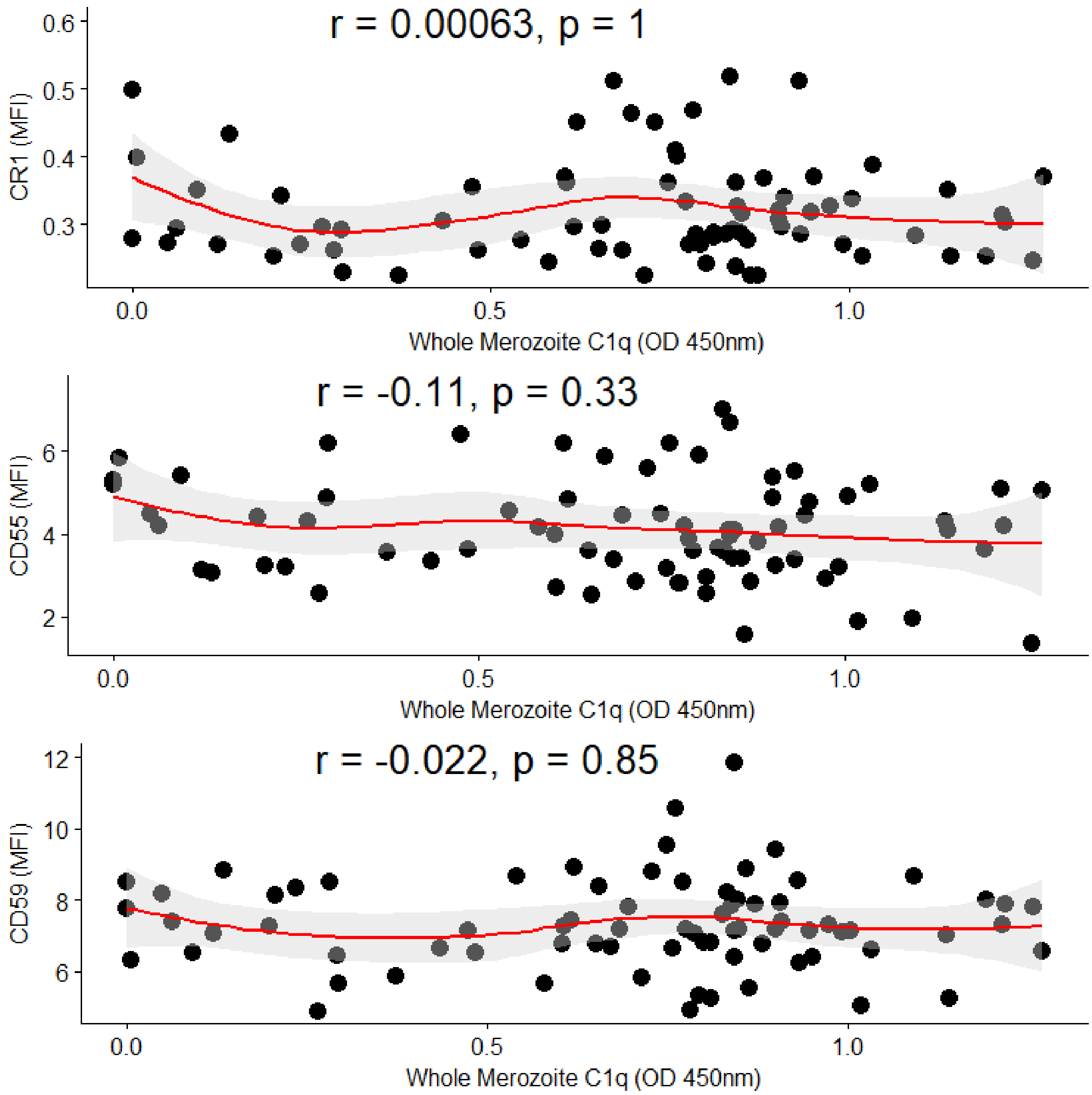

Figure S7. Relationship between CRPs expression and *P. falciparum* C1q fixing antibodies. Expression of CR1, CD55, and CD59 was correlated with absorbance of C1q fixing antibodies targeting purified *P. falciparum* whole merozoite (n = 76). Spearman's correlation coefficient is indicated. Red line indicates Loess non-parametric regression and gray band indicates 95% confidence interval..

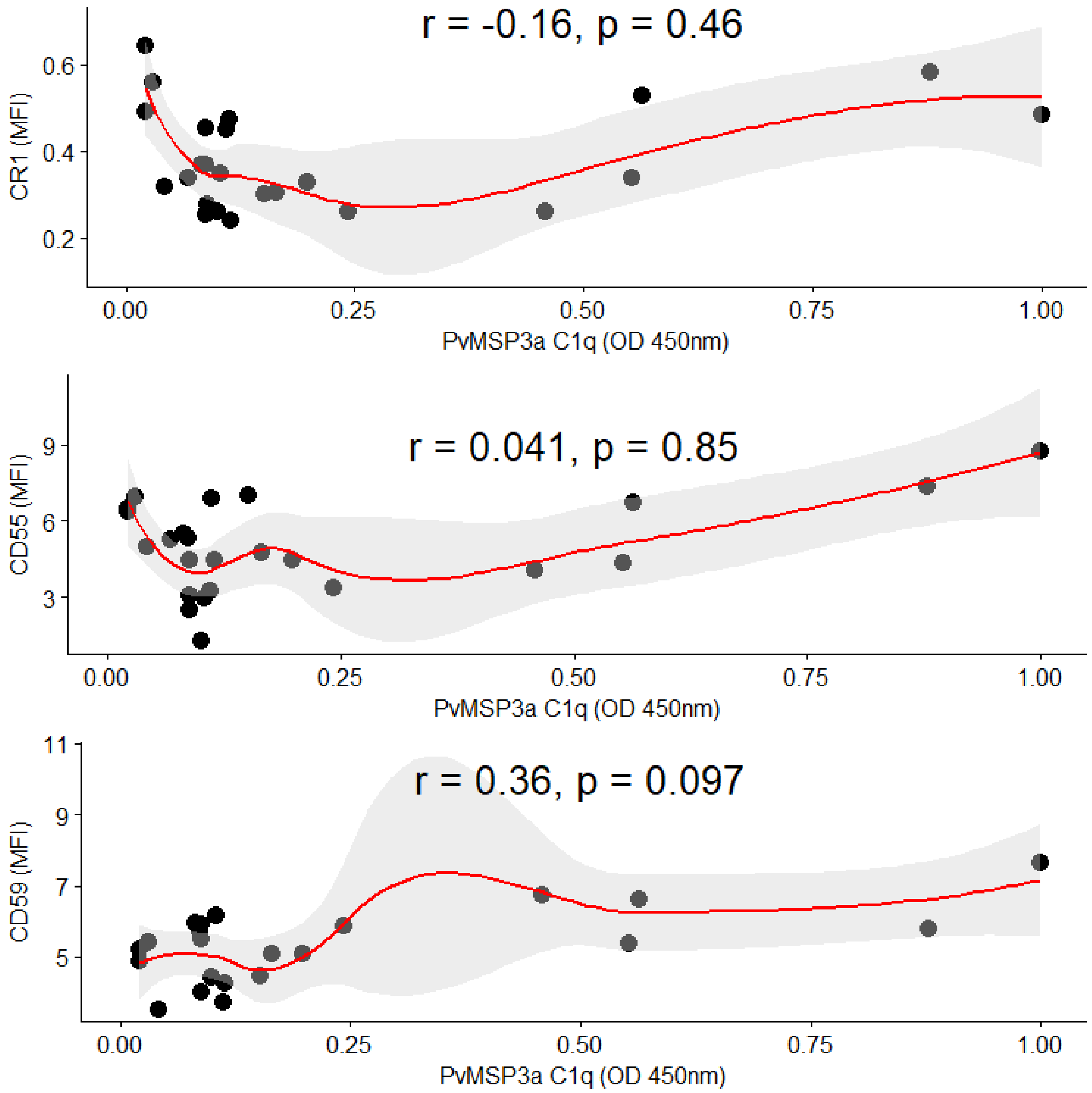

Figure S8. Relationship between CRPs expression and *P. vivax* C1q fixing antibodies. Expression of CR1, CD55, and CD59 was correlated with absorbance of C1q fixing antibodies targeting *P. vivax* MSP3α (n = 24). Spearman's correlation coefficient is indicated. Red line indicates Loess non-parametric regression and gray band indicates 95% confidence interval..

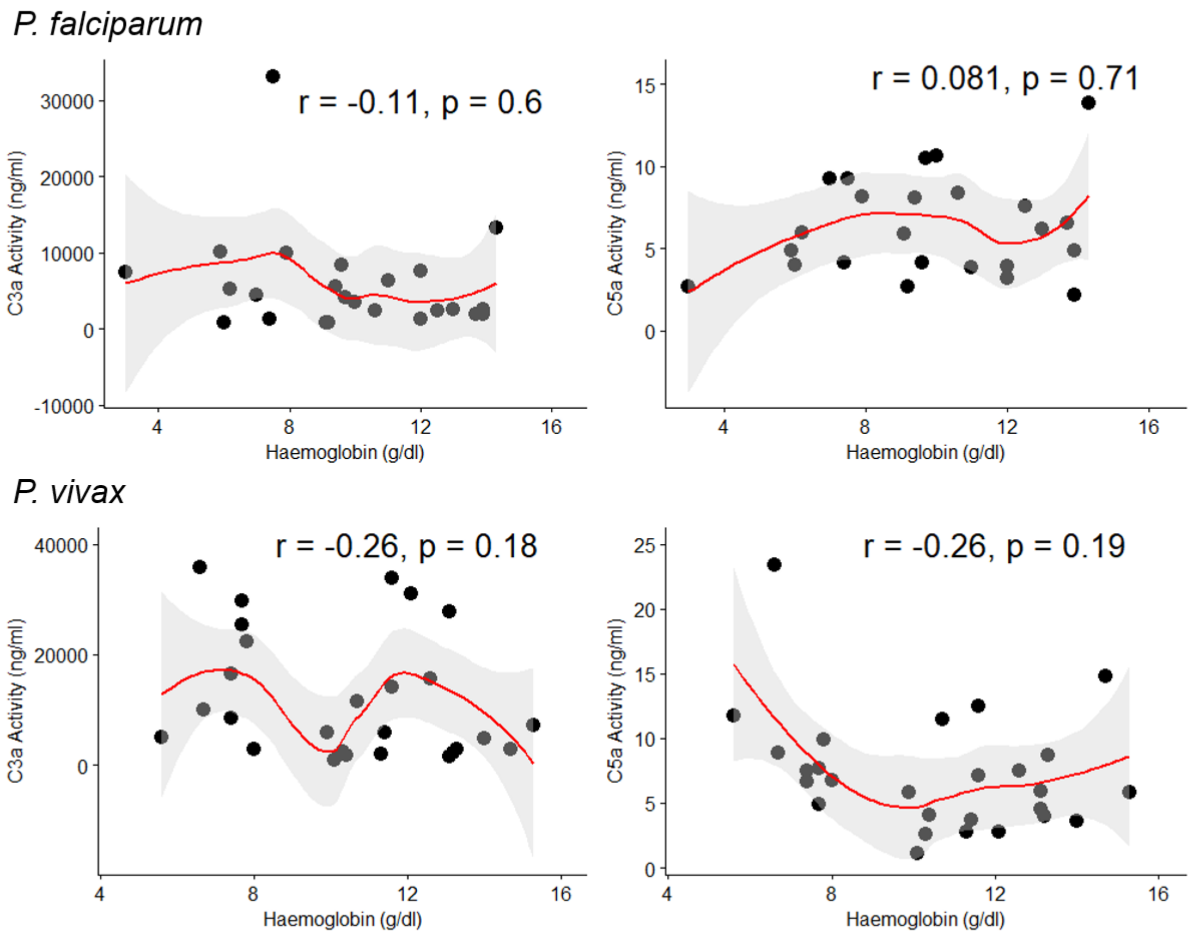

Figure S9. Relationship between complement activity and haemoglobin level in *P. falciparum* and *P. vivax* infected patients. Level of complement C3a and C5a was correlated with haemoglobin level (*P. falciparum* – C3a/C5a, n = 24, *P. vivax* – C3a/C5a, n = 27). Spearman's correlation coefficient is indicated. Red line indicates Loess non-parametric regression and gray band indicates 95% confidence interval..
